# Acoustic Emission Precursors in Pile-Reinforced Loess Landslides: A New Early-Warning Signals Identification Approach

**DOI:** 10.3390/s25247472

**Published:** 2025-12-08

**Authors:** Suya Zheng, Wei Yang, Tong Zhao, Xunchang Li, Zheng Lu

**Affiliations:** 1 College of Geological Engineering and Geomatics, Chang’an University, Xi’an 710054, China; 2023126120@chd.edu.cn (S.Z.); sleep_lin@126.com (T.Z.); dcdgx12@chd.edu.cn (X.L.); 2 State Key Laboratory of Loess Science, Xi’an 710054, China; 3 Key Laboratory of Western China’s Mineral Resources and Geological Engineering, Ministry of Education, Xi’an 710054, China; 4 Key Laboratory of Ecological Geology and Disaster Prevention, Ministry of Natural Resources, Xi’an 710054, China; 5 Northwest Electric Power Design Institute Co., Ltd. of China Power Engineering Consulting Group, Xi’an 710054, China; luzheng@nwepdi.com

**Keywords:** landslides, monitoring and early warning, acoustic emission, Kaiser effect

## Abstract

Monitoring landslide displacement and anti-slide pile damage is critical for assessing the stability of progressive loess landslides. To address the challenge of capturing precursor information for loess landslide instability under anti-slide pile reinforcement, this study systematically investigates the damage evolution process of slides (through their “slide-stability-reslide” cycles) and anti-slide piles under acoustic emission (AE) monitoring. Cyclic loading tests were employed to simulate the movement of progressive loess landslides. Based on the core causal logic that “slide displacement induces pile damage, damage generates AE signals, and signals invert displacement status”, a laboratory-scale physical model was designed to simultaneously monitor slide displacement, pile stress, and AE signals. The research results indicate that the dominant frequency and amplitude of AE signals are significantly correlated with slide displacement: with cyclic loading, both the dominant frequency and amplitude exhibit a “low → high → low” characteristic, corresponding to “low/medium-frequency low-amplitude”, “medium/high-frequency medium-high-amplitude” and “low-frequency medium-high-amplitude” signals in the three stages of slide deformation, respectively. The Kaiser and Felicity effects effectively monitor pile damage, and the decrease in Felicity ratio serves as a precursor for landslide early warning. Research results can provide a new methodological framework for early warning systems in pile-reinforced loess landslides.

## 1. Introduction

**Figure 1:**
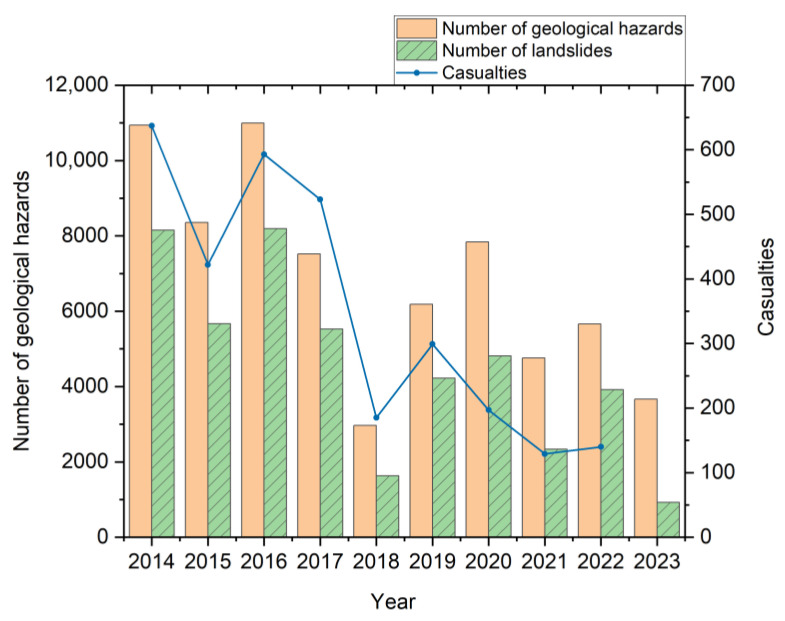
Statistics of landslide disasters in China in recent years.

Global environmental changes and intensified human activities have elevated landslides to a leading geological hazard [1,2,3]. In China, landslides accounted for 53% of all geological hazards in 2023, as shown in Figure 1, with 132,000 identified sites. These events pose severe threats to human safety, infrastructure [4,5], and ecosystems. Considering the substantial cost advantage of prevention over post-disaster recovery [6], establishing effective monitoring and early warning systems is crucial [7]. Landslides are broadly categorized by material composition into: (1) progressive loess landslides—characterized by clay/silt compositions, low shear strength, and rainfall-triggered gradual movements; (2) catastrophic rock landslides—involving intact/jointed rock masses that fail abruptly after prolonged stability [8]. Their spatial distribution and frequency vary regionally [9,10].

Landslides, defined as the gravitational downslope movement of rock and soil masses [11], are primarily identified through deformation and displacement monitoring [12]. Their initiation stems from progressive failure along sliding surfaces, making subsurface deformation monitoring crucial for early warning systems [13]. Traditional surface monitoring techniques (e.g., GNSS, InSAR, LiDAR) can achieve landslide risk assessment [14] but have limitations in cost, spatial coverage, and operational complexity. More critically, most of these monitoring methods cannot directly detect subsurface deformations—key precursors to catastrophic failures in real time [14]. This has driven research toward deep-seated monitoring using Shape Accel Arrays (SAA) and inclinometers, though these methods remain constrained by limited range, fragility, and high costs [15]. Recent advances highlight AE technology as a promising alternative. AE signals, generated by interparticle friction during slide displacement [16,17], have a quantitative correlation with deformation. To enhance signal detection, metal tube waveguides (passive for rock landslides [18]; active for soil landslides [19]) have been applied to the amplification of displacement-induced AE. The ringing count, a key AE parameter, exhibits a mathematical relationship with landslide velocity [20]. Active waveguide AE systems have been successfully deployed in soil landslides across the UK, China, and Canada [21], with studies confirming consistent correlations between AE signals (e.g., ringing count, energy, spectral changes) and displacement patterns [21,22,23]. These findings underscore the sensitivity, reliability, and real-time monitoring potential of AE technology in predicting slide instability.

Due to the complex mechanical properties of soil, there are significant challenges in the signal processing of AE from active waveguides. In China, where soil landslides represent approximately 70% of all cases [24], active waveguide technology helps overcome weak AE signal issues. Nevertheless, the intricate nature of soil-generated AE signals limits its broader application in landslide monitoring [25]. Field conditions exacerbate these challenges. Rainfall and anthropogenic activities trigger cyclic slide behavior (“slide-stability-reslide cycles”), rendering displacement-based stability assessments inadequate. Although anti-slide piles—favored for their construction versatility [26]—enhance slide stability, they experience structural degradation during slide deformation. Importantly, concrete pile failure produces measurable AE signals [27], revealing promising monitoring opportunities. AE technology has been proven effective in concrete damage evaluation [28,29,30], demonstrating capabilities to: quantify material strength degradation, monitor crack propagation and spatially localize fracture locations. These findings substantiate AE monitoring as a robust diagnostic tool for correlating slide deformation patterns with the structural integrity of anti-slide pile.

This study investigates progressive loess landslides through physical modeling, with a focus on slide displacement and anti-slide pile performance. AE sensors are installed on piles to monitor: (1) pile mechanical behavior and damage evolution under cyclic loading; (2) correlations between slide displacement and AE signal characteristics. The research aims to establish the relationship between AE signal variations and pile failure mechanisms during landslide movement, thereby identifying potential precursors of slide instability.

## 2. Materials and Methods

### 2.1. Experimental Setup

The experimental design followed three similarity theorems, with similarity conditions derived through equation and dimensional analysis [31]. Given site constraints, a geometric scaling factor (C_L = 20) was adopted, yielding a model container with a length of 4.3 m, width of 2.5 m, and height of 3 m. Table 1 summarizes the derived similarity relationships for other physical parameters. The overall frame of the model box is welded with square steel and channel steel, with glass panels on the four sides and the backfilled with wood panels and plastic film. Given that the main research objective of this study is to explore the failure effect of slide displacement on anti-slide piles, a double-layer plastic film with lubricant applied in between was selected to simulate the sliding zone.

**Figure 2:**
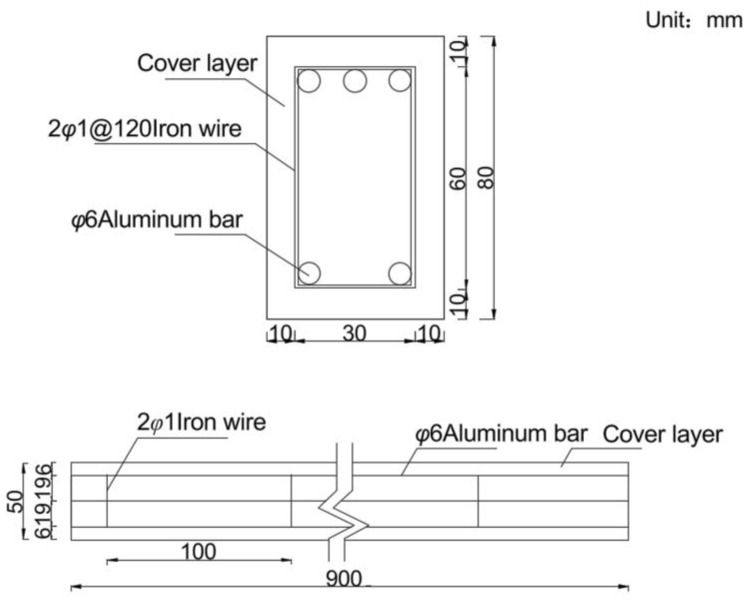
Schematic diagram of anti-slide pile reinforcement.

The anti-slide pile design followed the Design Specification for Anti-sliding Piles of Improvement Engineering, with an embedded length of 1/2–1/3 of the total pile length for soil/soft rock conditions. The model pile was 90 cm in total length (45 cm for embedded section and 45 cm for stress section), with cross-sectional dimensions of 0.8 cm in length and 0.5 cm in width, and 42.5 cm spacing between piles. The anti-slide pile was fabricated as a precast gypsum-aluminum rod composite model. Based on similarity principles, aluminum rods were employed to simulate steel reinforcement, while gypsum (with a mix ratio of 1:0.5:0.1 for gypsum: water: cement) represented concrete. The reinforcement configuration is illustrated in Figure 2.

**Figure 3:**
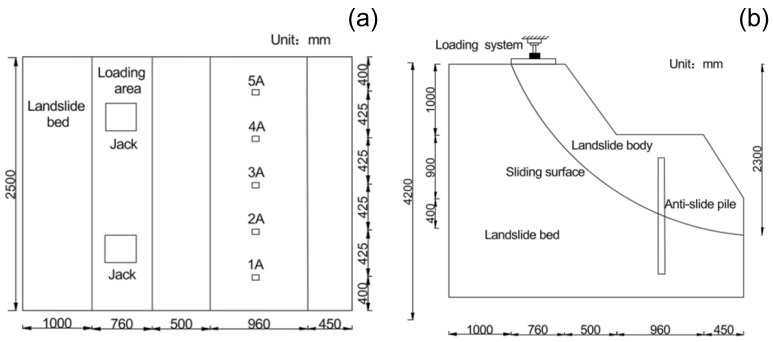
Structural configuration of the experimental model. (**a**) Vertical view. (**b**) Left view.

**Figure 4:**
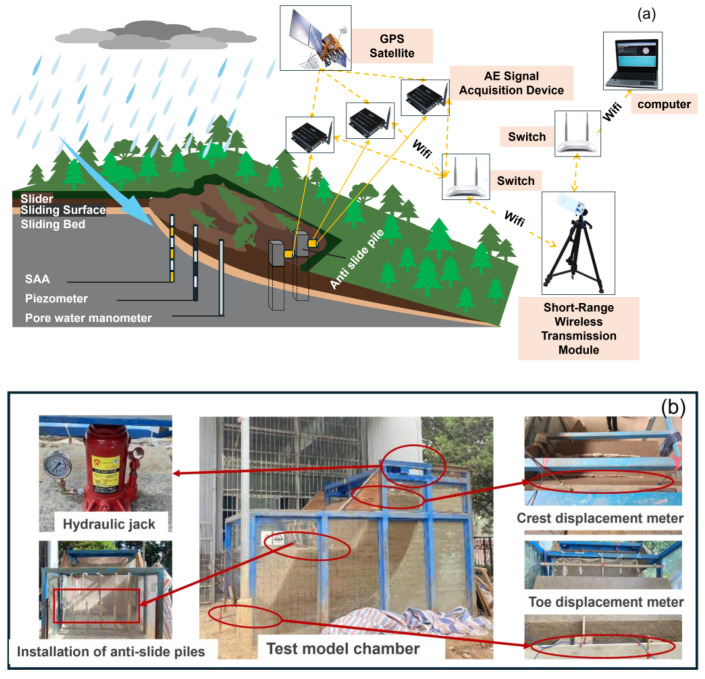
Conceptual diagram and physical realization of the landslide model. (**a**) Conceptual diagram of landslide mechanisms. (**b**) Physical landslide model.

The sliding bed material was prepared using compacted loess, achieved by first adjusting the water content to optimum levels, then placing and compacting in 200 mm layers to a final thickness of 150 mm per layer. A double-layer plastic film simulated the sliding surface with a circular-arc geometry. Based on the second similarity theorem applied to the elastic model, the pile reinforcement scheme consisted of 5 Φ6 aluminum longitudinal bars and Φ1 wire stirrups at 125 mm spacing, with 10 mm concrete cover. The experimental setup and simulated landslide geometry are shown in Figure 3 and Figure 4, respectively.

### 2.2. Loading System and Protocol

The experimental loading protocol was designed to systematically reproduce the characteristic slide-stability-reslide behavior of landslides [32,33] through controlled cyclic loading. The loading system incorporated two 15-ton hydraulic jacks equipped with pressure gauges, integrated with the top beam of model box and a reinforced loading plate. The hydraulic jacks were positioned between the support I-beam and the upper surface of the landslide body, enabling precise vertical load application through the reaction frame while simultaneously monitoring displacement responses.

**Figure 5:**
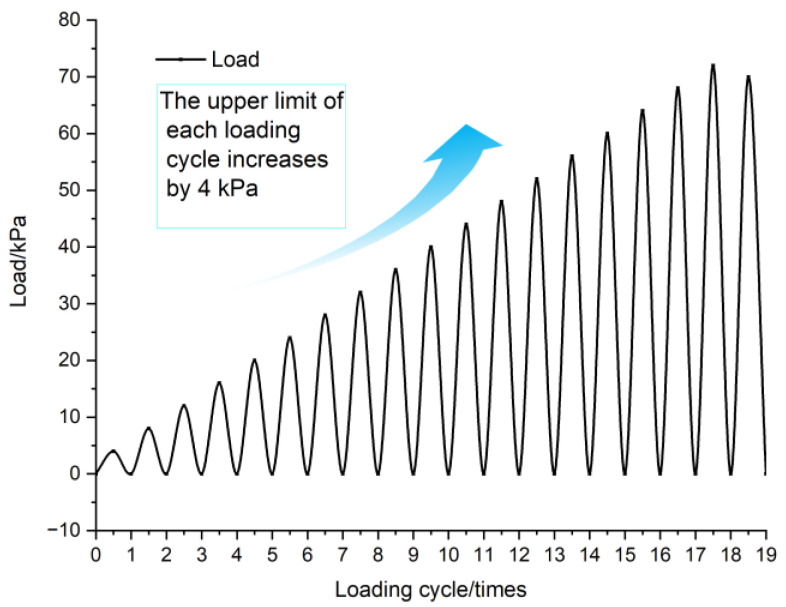
Cyclic staged loading.

The cyclic loading procedure involved progressively increasing the load limit by 4 kPa per cycle. During each cycle, AE, earth pressures, and displacements were recorded. Upon reaching stability, the load was gradually reduced before initiating the next cycle. This process continued until landslide failure occurred. Slide surface deformations were continuously photographed to analyze failure patterns. The loading sequence is shown in Figure 5.

### 2.3. Instrumentation and Measurements

#### 2.3.1. AE Monitoring System

**Figure 6:**
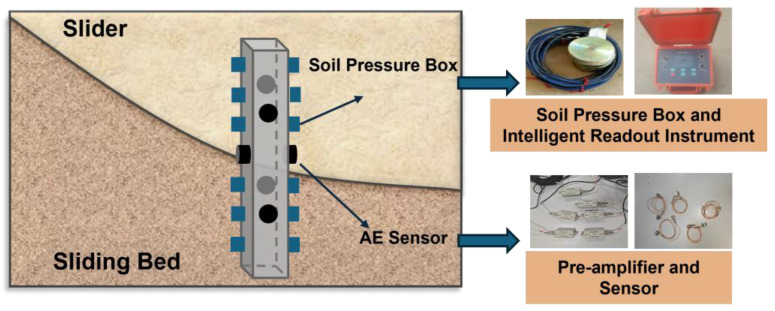
Layout of monitoring instrumentation.

AE monitoring was implemented to capture damage signals from anti-slide piles. Studies have shown that broadband sensors are suitable for monitoring concrete damage. Therefore, this study selects AE sensors with a resonant frequency of 80 kHz and a frequency response range of 5 kHz to 260 kHz. The selected sensor model can well cover the signal range generated by the damage of anti-slide piles. Six sensors were embedded within the anti-slide pile body, with two sensors each positioned at 30 cm, 45 cm, and 60 cm from the pile top. This symmetrical arrangement ensured comprehensive signal acquisition during the pile failure process. The sensor configuration is illustrated in Figure 6.

#### 2.3.2. Earth Pressure Measurement

Vibrating wire earth pressure cells were installed on both sides of the anti-slide pile to measure lateral earth pressures. Six pressure cells were embedded in vertical alignment on each face of the pile, with their sensing surfaces facing the loading surface and parallel to the pile surface. Earth pressure was automatically recorded using an intelligent readout instrument.

#### 2.3.3. Strain Measurement

The 120-2AA strain gauges were selected according to the size of the aluminum rods. Prior to installation, the rod surface was prepared by sanding with abrasive paper followed by alcohol cleaning. The gauges were then bonded using specialized adhesive. All strain gauges were connected to the TST3826F-L static strain acquisition system before pile installation, with initial zero-point readings recorded for each gauge.

#### 2.3.4. Slide Displacement Monitoring

To monitor slope displacement during loading, two dial displacement gauges (20 cm range, 0.1 mm accuracy) were installed at the slide crest and toe, respectively. Displacement measurements were automatically recorded using an intelligent readout instrument.

## 3. Experimental Results

**Figure 7:**
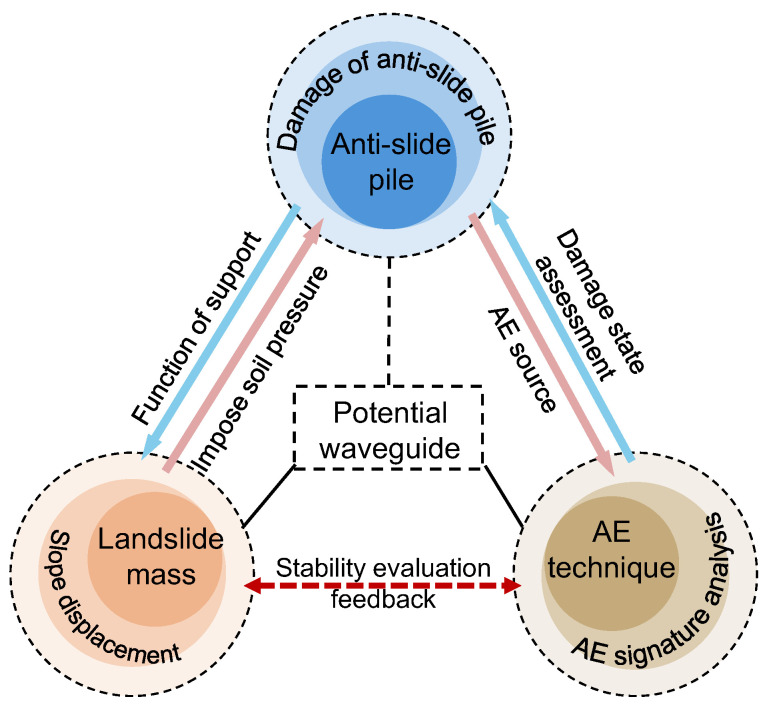
Correlation among landslide, anti-slide pile and AE technique.

The damage and failure of anti-slide piles originate from slide displacement, where the resulting forces induce progressive pile failure. And AE characteristics vary significantly with different damage levels. Therefore, as illustrated in Figure 7, a quantitative relationship between slide displacement, pile damage progression, and AE patterns can be established. Among the three relationships, the anti-slide pile, as a potential waveguide pipe, serves as the link for landslides and AE signals. Through the AE signal analysis, the failure of the pile can be characterized, and then the state of the landslide mass can be indirectly deduced, achieving the early identification of landslides.

### 3.1. Slide Behavior Analysis

#### 3.1.1. Displacement Characteristics

Field monitoring studies and failure mechanism analyses demonstrate that most landslide deformation processes follow a progressive pattern [34] and exhibit characteristic three-stage evolution [35,36,37]. The complete deformation process typically consists of initial deformation, stable deformation, and accelerated deformation stages.

**Figure 8:**
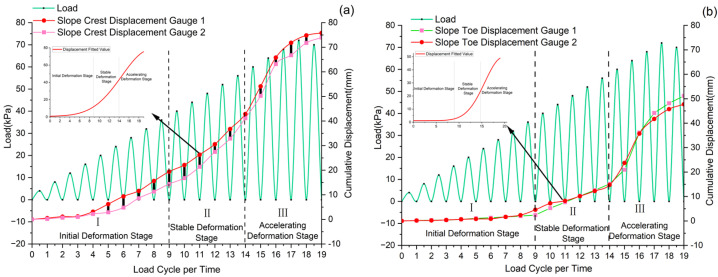
Time-history response of load versus displacement. (**a**) Vertical displacement evolution at the slide crest. (**b**) Horizontal displacement evolution at the slide toe.

Displacement gauges are mainly used to monitor the changes in vertical displacement at the slide crest and horizontal displacement at the slide toe. As shown in Figure 8, the temporal variations in load and displacement during cyclic loading tests reveal several key findings. With increasing cyclic loads, the slide experiences progressive cumulative displacement, where the crest displacement significantly exceeds that at the toe. This displacement pattern closely matches the established three-stage failure model [38,39], while the observed displacement differential between crest and toe provides clear evidence of the anti-slide pile’s effectiveness in slide stabilization.

**Figure 9:**
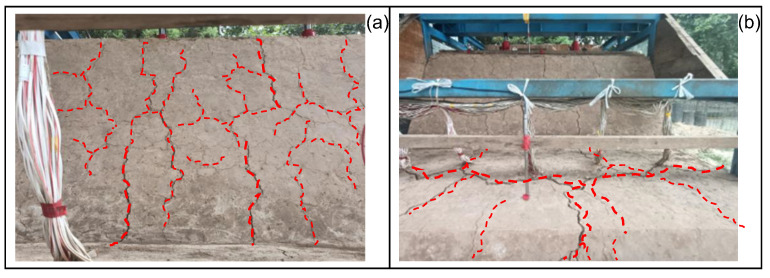
Slide deformation state diagram. (**a**) Secondary slide surface cracking at 16 kPa loading. (**b**) Primary berm cracking at 40 kPa loading.

The experimental results demonstrate three characteristic deformation stages under progressive loading. During initial loading (0–50% peak load), measured displacements at both slide crest and toe remain minimal (<2 mm), with no observable surface cracking. This reflects effective stress transfer through the compacted soil-pile system. The transitional stage (50–80 peak load) exhibits accelerated displacement rates, particularly at the crest (4.69 mm/cycle at the crest vs. 2.54 mm/cycle at the toe), accompanied by developing surface cracks (Figure 9a). Final loading stages (80–100% peak load) show dramatic displacement increases (crest: 6.6 mm/cycle; toe: 6.8 mm/cycle) and progressive crack propagation to 20 mm maximum width (Figure 9b), while pile resistance maintains a 48% reduction in toe displacement rates compared to uncontrolled conditions (slide crest). Internal damage in anti-slide piles cannot be visually inspected during service. The soil exerts forces on the pile body, and soil displacement can lead to surface failure. Therefore, there is a deterministic correlation between pile damage and soil displacement.

#### 3.1.2. Correlation Characteristics Between AE Signal and Slide Displacement

**Figure 10:**
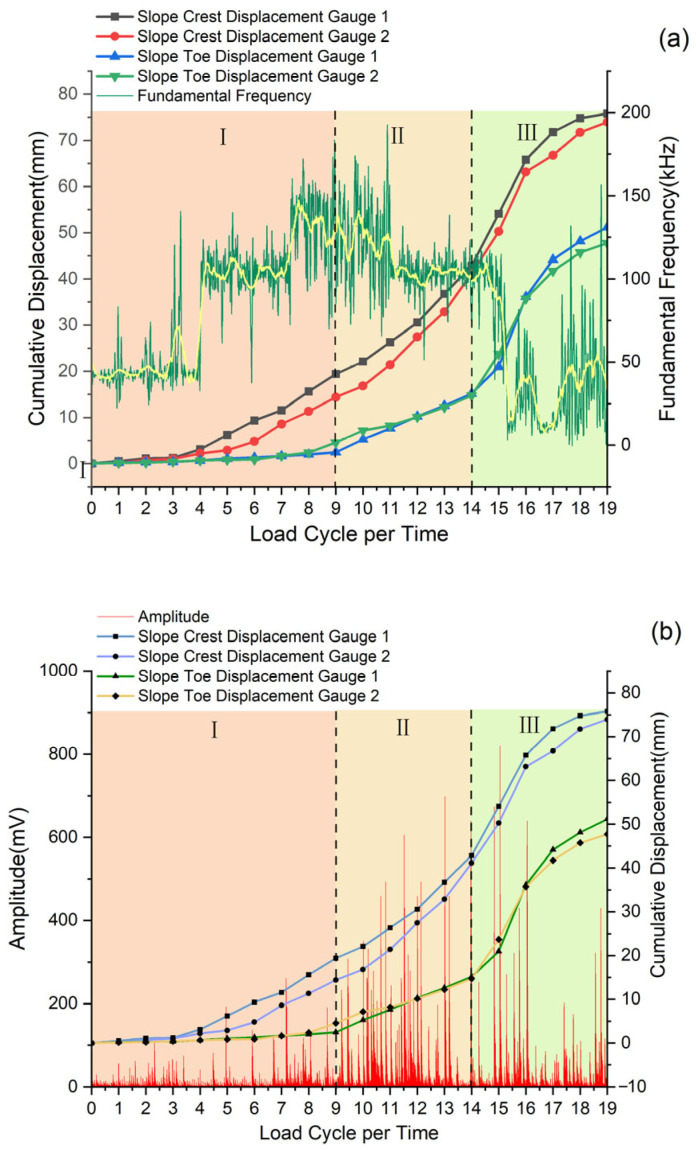
Time history response of frequency-amplitude-displacement. (**a**) Time history response of fundamental frequency-displacement. (**b**) Time history response of amplitude-displacement.

AE signal parameters effectively indicate landslide instability [40]. Spectral analysis reveals distinct frequency characteristics corresponding to different damage types. The Hilbert-Huang transform is used to extract dominant frequency and amplitude per unit time [41], which have a correlation with cumulative displacement, as shown in Figure 10.

Figure 10a illustrates the relationship between the dominant frequency of AE signals and displacement variation, with the dominant frequency showing a “low → high → low” trend. AE sensors were mounted on the anti-slide pile shaft, and the dominant frequency signals mainly characterize the type of pile damage. In the first stage of slide displacement, the signals are predominantly low and medium frequency, indicating slow deformation inside the pile. During this period, the slide undergoes gradual displacement in the soil compaction stage, continuously filling the existing gaps between soil particles, and the pile begins to bear the landslide thrust with slow internal deformation. In the second stage of slide displacement, the signals are mainly medium and high frequency, derived from particle collision and friction [42], as the slide enters the stable deformation stage. External loads and the slide’s self-gravity act on the pile, leading to microcracks inside the pile. In the third stage of slide displacement, the signals are mainly low frequency, corresponding to the formation of through-cracks in the pile that cause it to lose its retaining capacity, resulting in accelerated slide displacement and ultimate instability.

As shown in Figure 10b, the variation in AE amplitude signals is significantly correlated with the change in cumulative displacement. When the slide displacement accelerates, the dominant frequency amplitude signals begin to fluctuate drastically, accompanied by a large number of medium and high-amplitude signals. When the slide displacement reaches its maximum rate (i.e., the maximum slide of the displacement curve in the figure), the dominant frequency amplitude also peaks. At this point, the slide displacement mutates, large-scale cracks appear inside the slide, and high-amplitude signals emerge accordingly.

Studies have shown that high-amplitude signals are usually associated with low-frequency signals [43]; thus, low-frequency and high-amplitude signals to a certain extent reflect the process of slide instability and failure. In the third stage of the landslide, AE signals exhibit medium-high amplitude and low frequency, indicating that cracks have formed inside the anti-slide pile, which can no longer effectively perform its retaining function, leading to accelerated slide displacement and instability.

Displacement variation simply reflects the slide instability process during the entire loading cycle, but the displacement changes at the slide crest and toe are inconsistent, indicating non-uniform deformation across different slide positions. As shown in Figure 10b, toe displacement well characterizes the overall slide deformation. However, slide displacement does not increase significantly even in the second stage, only rising rapidly upon reaching the failure point in the third stage. In contrast, the dominant frequency amplitude signals in the second stage already exhibit low-frequency and high-amplitude characteristics with increased density of medium-high amplitude signals, indicating impending slide instability. Thus, changes in dominant frequency and amplitude can serve as precursors to identify the trend of slide instability and failure.

### 3.2. Anti-Slide Pile Performance

#### 3.2.1. Mechanical Response Analysis

**Figure 11:**
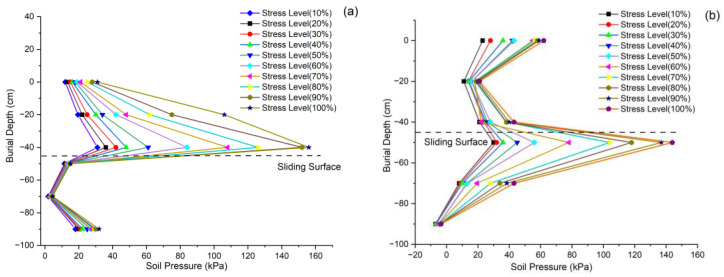

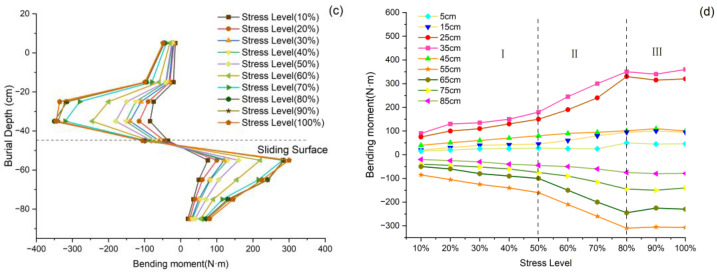
Earth pressure and bending moment distribution along pile shaft. (**a**) Earth pressure behind pile. (**b**) Earth pressure in front of pile. (**c**) Bending moment distribution along pile shaft. (**d**) Bending moment distribution along pile shaft.

Earth pressure measurements obtained from embedded strain gauges demonstrate characteristic pressure distribution patterns around the anti-slide pile during cyclic loading (Figure 11). Above the slip surface, the earth pressure behind the pile increases progressively with depth, reaching maximum values adjacent to the slip surface, while the pressure in front of the pile decreases gradually. Below the slip surface, the rear pressure diminishes with increasing depth, whereas the frontal pressure exhibits a marked increase, including at the pile base. This pressure distribution pattern, characterized by predominant rear pressure above the slip surface and frontal pressure concentration below it, corresponds to established pile loading behavior in slide stabilization systems [44]. The bending moment distribution analysis reveals that the peak bending moment consistently occurs in proximity to the sliding surface (Figure 11c). The test results indicate that the maximum bending moment of the anti-slide pile occurs at 15 cm above and 10 cm below the sliding surface. Near the sliding surface, the bending moment approaches zero, suggesting peak shear forces at this location. Furthermore, the actual pivot point of the pile coincides approximately with this position. Notably, the bending moment magnitude at all measurement points exhibits a positive correlation with applied load intensity (Figure 11d). Furthermore, the most rapid rate of bending moment variation was observed during the secondary stage of slide displacement.

#### 3.2.2. AE-Damage Evolution Characteristics

**Figure 12:**
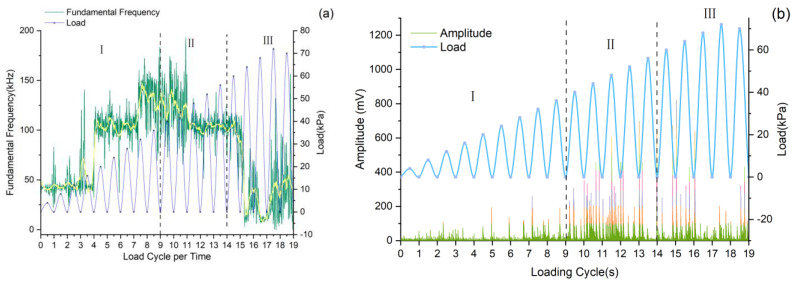
Fundamental Frequency, amplitude and load curve. (**a**) Fundamental Frequency and load curve. (**b**) Amplitude and load curve.

The evolution of AE signal characteristics during progressive loading (Figure 12) reveals three distinct stages: (1) Initial loading stage: Dominant low-frequency, low-amplitude signals indicate gradual compaction of pre-existing fractures within the pile, with minimal energy release. (2) Intermediate stage: The appearance of intermediate- and high-frequency signals, accompanied by increasing amplitude in high-frequency components, suggests particle interactions and microcrack formation [42]. (3) Final stage: Low-frequency, high-amplitude signals demonstrate the development of through-going fractures, consistent with macroscopic failure mechanisms. These observations align with established AE theory [43], where low-frequency signals correspond to large-scale crack propagation (long damage timescales), while high-frequency components reflect particle-scale interactions (short damage timescales).

**Figure 13:**
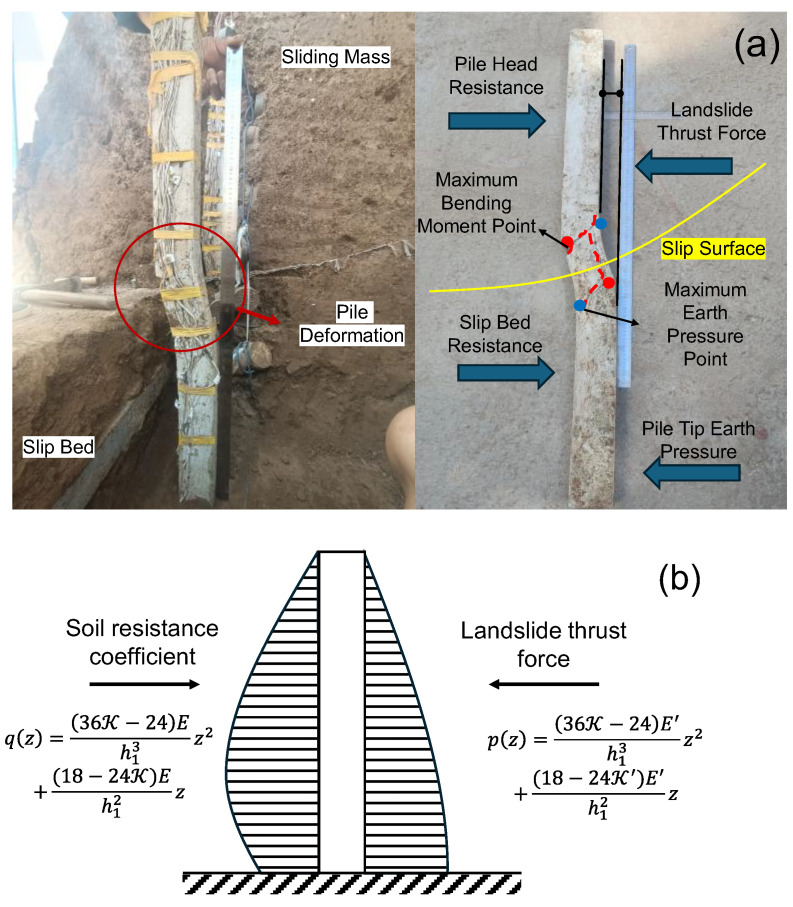
Pile body failure and deformation situation. (**a**) Deformation and failure of pile body. (**b**) Mechanical analysis of pile structure.

The deformation characteristics and failure mechanism of the anti-slide pile are presented in Figure 13. The pile exhibits forward tilting above the slip surface due to combined landslide thrust and basal resistance, while developing backward displacement tendencies below this interface. Notably, the pile movement direction opposes the sliding mass displacement below the slip surface, resulting in reduced earth pressure at the pile base. A prominent macroscopic fracture near the slip surface correlates with the observed low-frequency, high-amplitude AE signals, suggesting the structural failure of the anti-slide pile is a possible source of AE.

#### 3.2.3. Localization Characteristics of AE Signals in Anti-Slide Piles

The landslide thrust induces AE activity within anti-slide piles, corresponding to three distinct damage mechanisms: crack compaction, initiation, and propagation. Three-dimensional AE source localization verifies these damage processes and establishes their correlation with dominant frequency and amplitude characteristics.

**Figure 14:**
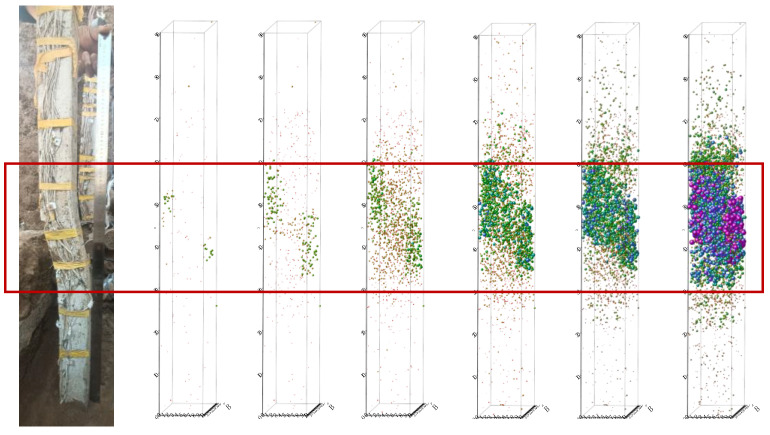
Spatial positioning evolution diagrams of AE events under different loads.

Figure 14 displays AE events as colored particles, where particle size corresponds to event amplitude and rupture scale. Color gradation (green to purple) indicates signal intensity from weak to strong. The spatial distribution and density of these particles illustrate crack initiation and propagation within the pile, with higher particle concentrations indicating more intensive fracture activity. Figure 14 presents a sequential representation of AE event location maps within the anti-slide pile, showing (from left to right): the actual failure condition, the first cyclic load, the fourth cyclic load, the eighth cyclic load, the 11th cyclic load, the 15th cyclic load, and the 19th cyclic load. The spatial distribution of AE events corresponds well with the deformation pattern observed under actual failure conditions. This correlation demonstrates that cyclic loads applied at the slide crest propagate through the slide mass and induce internal deformation in the pile. These findings provide further evidence for the mechanical interaction between the slide mass, anti-slide pile, and the applicability of AE monitoring technology (Figure 7).

## 4. Discussion

### 4.1. Damage Evolution Characteristics Within Anti-Slide Piles

To further elucidate the interrelationships among slide deformation, anti-slide pile failure, and AE signals, we systematically classified the slide movement stages and conducted a comprehensive analysis of anti-slide pile fracture signals, slide displacement patterns, and AE spectral characteristics at each stage.

**Figure 15:**
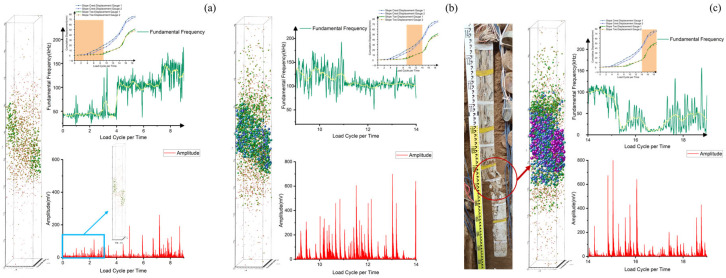
Coupling analysis between AE signatures and pile damage evolution. (**a**) AE events during initial stage. (**b**) AE events during accelerated deformation stage. (**c**) AE events during failure stage.

Figure 15a illustrates initial pile damage development during early slide displacement. Two AE event types are identified: (1) high-amplitude, large-particle events concentrated near the sliding surface (corresponding to Figure 11a,b), suggesting fracture initiation; and (2) low-amplitude, small-particle events distributed throughout, reflecting soil compaction and fracture closure. Spectral analysis indicates predominantly low-frequency signals at this stage. Despite accelerating slide displacement, the pile maintains load-bearing capacity with limited damage, preserving slide stability.

Figure 15b depicts pile damage during the second stage of slide displacement. Blue signal particles gradually emerge in the positioning map, increasing in volume and accumulating, indicating crack propagation within the pile. Concurrently, medium- and high-amplitude AE signals appear with step-type characteristics, confirming progressive crack deepening [45]. The accumulated strain energy from the previous stage is gradually released, while AE signals shift to mid- and high frequencies, suggesting internal particle collisions and friction. At this stage, the pile enters a new stage of damage, awaiting further energy release.

Figure 15c illustrates pile damage during the third stage of slide displacement. A significant number of blue and purple signal particles appear, with a sharp frequency drop and peak signal amplitude. Low-frequency, high-amplitude AE signals dominate, triggering another strain energy release and large-scale crack events. Consequently, slide displacement accelerates, pile stress surges, and structural integrity deteriorates. The pile loses its load-bearing capacity, leading to macroscopic slide cracking and eventual instability.

The analytical results demonstrate a definitive correlation between damage of anti-slide pile, slide movement patterns, and signal characteristics of AE. As slide deformation progresses, the internal fracture signals within the anti-slide pile exhibit continuous intensification, ultimately coalescing into macroscopic cracks. Correspondingly, the AE amplitude displays a step-type increase pattern, with particularly significant amplification during the secondary deformation stage. The subsequent massive occurrence and interconnection of internal fracture signals in the anti-slide pile indicate imminent structural failure. These findings suggest that the step-type characteristics of AE amplitude signals can serve as reliable precursors for slide instability. Further quantitative assessment of anti-slide pile damage conditions requires additional AE signal processing methodologies.

### 4.2. Kaiser and Felicity Effects in Stabilizing Piles Under Cyclic Loading

The Kaiser effect is typically applied to rock or concrete materials, where irreversible deformation of such materials is accompanied by the Kaiser effect in AE. During the initial stage with no obvious damage or the elastic stage of the material, significant AE activity occurs when the stress exceeds the historical maximum stress level; moreover, the harder the rock, the more pronounced the Kaiser effect. The Felicity effect is commonly used in the crack propagation stage of materials and can serve as an important indicator for damage monitoring [46]. These two effects represent the continuous variation in AE signal responses during the material damage evolution process. The Kaiser and Felicity effects are employed to characterize the irreversibility of anti-slide pile damage. In this study, AE signal ringing counts are extracted to calculate the cumulative ringing count rate, which can reflect the frequency of AE events during pile deformation [47]: an increasing count rate indicates the gradual occurrence of AE events, while a peak count rate corresponds to the rapid generation of AE signals. Therefore, the above two effects can be determined by analyzing the relationship between the current load and the historical peak load [48].

**Figure 16:**
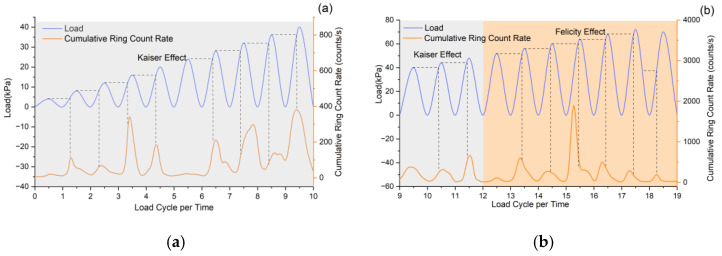
Characterization of Kaiser and Felicity effects in stabilizing piles under cyclic loading. (**a**) Kaiser effect observed within 0–10 loading cycles. (**b**) Felicity effect emerging after 12 loading cycles.

In Figure 16 (excluding cycle 19), dashed lines mark cumulative ringing count rate changes when loads reach previous peak levels. For the first twelve cycles, rate peaks occur at or right of these lines, showing that AE events surge when loads match or exceed prior maxima, signaling internal material changes. This pattern confirms concrete follows Kaiser effect behavior under cyclic loading, though not uniformly (e.g., cycle 6).

From the 1st to 12th loading cycles, the peak cumulative ringing count rate progressively shifted left of the dashed line. This demonstrates that AE signals peaked before reaching prior loading-cycle maxima, exhibiting Felicity effect characteristics opposite to the Kaiser effect [49]. The effect’s intensity correlated with material damage degree, indicating irreversible pile damage onset from the 13th cycle onward. This plastic deformation stage corresponds to slide displacement’s accelerated deformation stage, serving as a precursor to imminent slide failure.

### 4.3. Felicity Ratio of Stabilizing Piles Under Cyclic Loading

**Figure 17:**
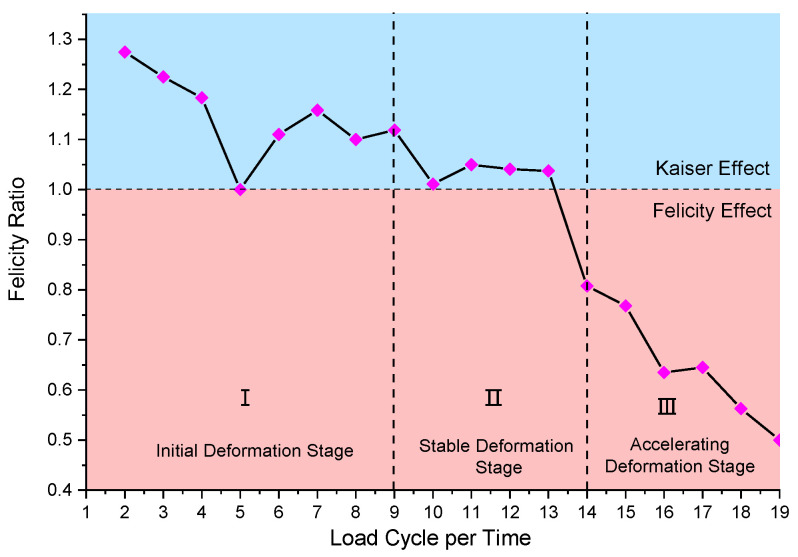
Felicity Ratio under cyclic loading.

To further investigate the correlation between the Felicity effect and material damage progression, the variation characteristics of the Felicity ratio (FR) are analyzed [50,51]. The FR serves as a reliable indicator of material damage severity and provides critical evidence for identifying material defects, with lower values corresponding to more pronounced damage. Previous studies have demonstrated that the FR of typical rock specimens ranges between 0.8 and 1.4, where 0.8 represents the threshold for complete rock failure [52,53]. Notably, the Kaiser effect dominates when FR ≥ 1, whereas the Felicity effect becomes apparent when FR < 1. Throughout the loading process, the FR exhibited minimal fluctuations within a narrow range during Kaiser effect dominance, showing an overall declining trend. In contrast, the emergence of the Felicity effect is consistent with a marked reduction in FR. Figure 17 illustrates these characteristic FR variations under cyclic loading conditions.

The damage of anti-slide pile accumulates with increasing loading cycles, as evidenced by the continuous FR reduction. During cycles 1–9, the FR remains greater than 1 while declining gradually at an average rate of 2.2%, corresponding to the slide’s initial deformation stage where the pile demonstrated elastic behavior (Kaiser effect) with recoverable deformation. A pronounced FR drop to 1.01 occurred at the 9th cycle. Cycles 10–13 maintained FR ≈ 1.02, marking the slide’s secondary deformation stage where internal cracks propagated within the pile. The 14th cycle witnessed a critical FR decline to 0.8, signaling irreversible plastic deformation in the pile, internal crack coalescence, progressive loss of retaining capacity, and eventual slide instability.

Experimental results and failure observations indicate that structural failure of the pile and slide instability conforms to FR < 1 at the 14th loading cycle. The consistent FR decline approaching unity during cycles 10~12 may serve as a precursor for landslide instability, providing valuable early warning information.

## 5. Conclusions

Through cyclic graded-loading model tests, this study investigates correlations between landslide displacement, pile damage, and AE signal characteristics, and establishes early-warning indicators. AE spectral and parametric analyses reveal their relationships with structural damage progression and slide movement. Key findings include:

1. Through the analysis of landslide displacement, the evolution of landslide can be divided into three distinct deformation stages: initial soil compaction, elastic-to-plastic transition, and plastic failure. Corresponding AE signal analysis showed the dominant frequency initially increased then decreased with displacement, particularly dropping to extremely low frequencies near instability. Simultaneously, high-amplitude signals increased progressively. These systematic AE parameter variations provide reliable precursors for slide failure prediction.

2. Soil pressure analysis and AE source localization reveal the progressive failure process of piles under cyclic loading. The transition from Kaiser effect (early-mid stage) to Felicity effect (late stage) quantitatively characterizes damage accumulation, with Felicity ratio variations serving as reliable slide instability precursors.

As an exploratory experiment, this study verifies the effectiveness of AE technology in monitoring landslide precursors and the damage evolution of anti-slide piles, demonstrating promising potential for landslide early warning applications. However, due to current experimental limitations, the influence of complex natural/anthropogenic factors (e.g., rainfall, earthquakes, excavation) on landslide and anti-slide pile is not considered. Future research should investigate AE characteristics under these complex field conditions.

## Figures and Tables

**Table 1:** Similarity relationship analysis for landslide model tests.

Types	Physical Parameter	Dimensionality	General Model	Practical Model	Proposed Model
Material properties	Stress σ	${FL}^{-2}$	$K_{\sigma }$	1	20
	Strain ε	-	1	1	1
	Elastic modulus E	${FL}^{-2}$	$K_{\sigma }$	1	20
	$\mathrm{Shear}\;\mathrm{modulus}\;G_{m}$	${FL}^{-2}$	$K_{\sigma }$	1	20
	Compressive strength R	${FL}^{-2}$	$K_{\sigma }$	1	20
	Cohesion C	${FL}^{-2}$	$K_{\sigma }$	1	20
Geometric characteristics	Length L	L	$K_{L}$	$K_{L}$	20
	Linear displacement Δ	L	$K_{L}$	$K_{L}$	20
	Angular displacement β	-	1	1	1

## Data Availability

The original contributions presented in this study are included in the article. Further inquiries can be directed to the corresponding author.
